# High carbohydrate intake from starchy foods is positively associated with metabolic disorders: a Cohort Study from a Chinese population

**DOI:** 10.1038/srep16919

**Published:** 2015-11-19

**Authors:** Rennan Feng, Shanshan Du, Yang Chen, Sining Zheng, Wei Zhang, Guanqiong Na, Ying Li, Changhao Sun

**Affiliations:** 1 Department of Nutrition and Food Hygiene, Public Health College, Harbin Medical University, Harbin, China

## Abstract

Starchy foods are the main sources of carbohydrates; however, there is limited information on their metabolic impact. Therefore, we assessed the association between carbohydrates from starchy foods (Carb-S) intakes and the metabolic disorders of metabolic syndrome (MetS) and hyperlipidemia. In this study, 4,154 participants from Northern China were followed up for 4.2 years. Carb-S included rice, refined wheat, tubers, and their products. Multivariable regression models were used to calculate risk ratios (RRs) for MetS and hyperlipidemia from Carb-S, total carbohydrates, and carbohydrates from other food sources (Carb-O). Receiver operating characteristic analysis was used to determine a Carb-S cut-off value. High total carbohydrate intake was associated with increased risks of MetS (RR: 2.24, 95% CI: 1.00–5.03) and hyperlipidemia (RR: 3.05, 95% CI: 1.25–7.45), compared with the first quartile. High Carb-S intake (fourth quartile) was significantly associated with MetS (RR: 1.48, 95% CI: 1.01–2.69) and hyperlipidemia (RR: 1.73, 95% CI: 1.05–3.35). No associations with Carb-O were observed. Visceral adiposity, triglyceride levels, and high-density lipoprotein cholesterol significantly contributed to the metabolic disorders. The Carb-S cut-off value was 220 g. Both high total carbohydrate and Carb-S intakes were associated with hyperlipidemia and MetS; Carb-S appears to contribute more to these disorders.

The relationship between diet composition and human health has been debated for more than half a century. The effects of high and low carbohydrate diets on metabolic disorders are not clear^1^. Based on recent evidence, low carbohydrate diets increased the risk for gestational diabetes mellitus in a pregnancy cohort study^2^ and for cardiovascular disease in a Swedish cohort study^3^. These diets were also positively associated with cancers and all-cause mortality^4^,^5^,^6^. Additionally, in prospective cohort studies, high carbohydrate diets were associated with increased incidence of stroke^4^ and diabetes in Chinese individuals^5^, but not in Caucasian^6^ or American subjects^7^. More confusingly, the total carbohydrate amount is not significantly associated with ischemic heart disease mortality in Asian populations^8^ and does not affect insulin resistance in healthy eumenorrheic women^9^. Based on the findings obtained from a meta-analysis of 7 prospective cohort studies, there is no association between high carbohydrate intake and stroke risk^10^. Faced with these inconsistent results, there is confusion regarding the decisions that are most beneficial to health in the daily diet.

In Asia, starchy foods represent the main source of dietary energy. According to the Chinese Nutritional Guidelines, carbohydrates should contribute 55–65% of the daily energy intake^11^, which is higher than that recommended in the USA (45–65%). Chinese diets are rich in starchy foods (e.g., refined grain, tubers, and their products), which are positively associated with increased risks of cerebrovascular diseases^12^, breast cancer, and type 2 diabetes mellitus (T2DM)^5^,^13^. Recently, a dose-response meta-analysis revealed that diets high in white rice are associated with an increased risk of T2DM, especially in Asian (Chinese and Japanese) populations^14^. Moreover, nondigestible and digestible carbohydrates differ in their glycemic index (GI) and glycemic load (GL)^15^. Starchy foods, including refined rice and wheat, have high GI and GL values. High GI/GL diets increase the risk of stroke, coronary heart disease, depression, and cancer^10^,^12^,^16^,^17^, while low GI/GL diets benefit human metabolism^18^.

It is not clear whether starchy foods, which are highly consumed by Asian populations^19^,^20^, contribute to a higher risk for metabolic disorders. In this study, we analyzed a cohort from 2008^21^ to evaluate the association between different carbohydrate sources and the incidence of metabolic disorders in China.

## Results

### Baseline characteristics of subjects based on carbohydrate intake from starchy foods

We excluded participants with metabolic syndrome (MetS); the demographic and biochemical characteristics of the remaining 2,734 participants are summarized in Table 1. There were no significant differences among the four quartiles in body mass index (BMI), waist circumastance (WC), diastolic blood pressure (DBP), blood glucose, insulin, high-density lipoprotein cholesterol (HDL-C), or prevalence of obesity, diabetes, or hyperlipidemia. There were, however, significant differences in age, gender, smoking tendency, systolic blood pressure (SBP), total cholesterol (TC), and serum triglyceride (TG) among the four groups.

### Associations between carbohydrate intake and hyperlipidemia and MetS

A total of 369 subjects were diagnosed with MetS during the 4.2-year follow-up. Table 2 shows that the risk ratios (RRs) of the highest and second highest quartiles for MetS were 1.44 (1.02–2.04) and 1.81 (1.29–2.55), respectively, compared to the first quartile of carbohydrates from starchy foods (Carb-S) without covariates in Model 1. In the multivariate regression model (Model 4), the combined RRs for the top and second quartiles versus the lowest quartile for MetS were 1.48 (1.01–2.69) and 1.89 (1.19–3.02), respectively.

For hyperlipidemia, a total of 2,252 participants were included in the analyses. High Carb-S intake was positively associated with an increased risk of hyperlipidemia. Compared to the lowest quartile, the RRs of the upper and second highest quartiles of Carb-S in Model 1 were 1.63 (1.15–2.32) and 1.47 (1.03–2.10), respectively. In Model 4, the combined RRs for the top and second highest quartiles versus the lowest Carb-S were 1.73 (1.05–3.35) and 1.91 (1.13–3.23), respectively (Table 2).

For MetS, we stratified the population according to the total carbohydrate intake. The RRs of the third and fourth groups were 1.59 (0.95–2.65) and 2.24 (1.00–5.03), respectively (Table 3). In this manner, we re-analyzed the risk factors for hyperlipidemia. Higher total carbohydrate intake increased the risk of hyperlipidemia (RR: 3.05, 95% confidence interval [CI]: 1.25–7.45; Table 3). There were no significant associations between carbohydrates from other food types (Carb-O) and the metabolic disorders (Table 4).

### Metabolic changes during the 4.2-year follow-up

During the 4.2-year follow-up, anthropometric and biochemical indices changed; visceral adiposity index (VAI) (P = 0.05) and TG (P = 0.04) increased and HDL-C decreased (P = 0.01) in all four quartiles of Carb-S. There were no changes in other anthropometric (BMI, WC, SBP, or DBP) or biochemical (fasting blood glucose [FBG], 2-hour post-load blood glucose [2h-PG], fasting insulin [F-insulin], 2-hour post-load insulin [P-insulin], or insulin resistance [HOMA-IR], TC) indices (Table 5).

### Optimal carbohydrate intake

The sensitivity and specificity of starchy foods in the diagnoses of MetS and hyperlipidemia are shown in Table 6. The area under the curve was 0.65 (95% CI: 0.62–0.68) for MetS and 0.68 (0.62–0.69) for hyperlipidemia. For MetS, the Carb-S threshold was 230 g with a Youden index of 13.41%, a sensitivity of 64.23%, and a specificity of 49.18%. For hyperlipidemia, the Carb-S threshold was 220 g, with a Youden index of 12.05%, a sensitivity of 64.27%, and a specificity of 47.78%. Sensitivity, specificity, and Youden indexes decreased when Carb-S was lower or higher than 220 g (Table 6).

### Association between high GI starchy foods and hyperlipidemia and MetS

In the multivariable adjusted regression model, high GI starchy foods were associated with significantly higher risks for hyperlipidemia (1.32; 1.03–2.17) and MetS (1.57; 1.01–2.42). However, there was no significant association between medium or low GI groups and risks of hyperlipidemia (0.93; 0.63–1.37) or MetS (0.76; 0.53–1.11; Table S1).

## Discussion

In this study, we analyzed the association between high carbohydrate intake from starchy foods and metabolic disorders. First, we confirmed that high total carbohydrate intake is positively associated with hyperlipidemia and MetS. Second, we found that high carbohydrate intake from starchy foods may contribute to the pathogenesis of these metabolic diseases, while there were no significant associations between the intake of other carbohydrates and metabolic disorders. During the follow-up period, visceral adiposity and serum TG increased, while HDL-C decreased in the high Carb-S group. Finally, we concluded that a daily consumption of 220 g of Carb-S protects against metabolic disorders in northern China.

Our findings suggest that Carb-S could contribute to the pathogenesis of metabolic disorders. Carbohydrate intake decreased HDL-C and increased TG levels. A human clinical trial reported that participants consuming high carbohydrate diets had lower HDL-C and higher TG and TC levels^22^. Low carbohydrate intake has the opposite effects because high fat diets tend to lower serum TG and LDL-C, increase HDL-C^23^, and promote weight loss^22^. This phenomenon may be partially explained by the activity of lipoprotein lipase (LPL) in hepatocytes and enterocytes. LPL mediates the catabolism of triacylglycerol-rich lipoproteins, unesterified cholesterol, and phospholipids. In mice, overexpressed LPL increases HDL-C levels. High-carbohydrate diets may inhibit LPL action, thereby inducing fatty acid production in hepatocytes^24^, increasing TG levels, and decreasing HDL-C levels.

Refined grains and tubers have high GI and GL^25^,^26^, inducing pathoglycemia and insulin resistance^27^, which are risk factors for hyperlipidemia through fat deposition and *de novo* lipogenesis^28^. Our findings revealed that the high GI group had significantly higher risks of hyperlipidemia and MetS. No significant associations were obtained between the medium or low GI groups and hyperlipidemia or MetS. High GI diets increase body fat mass and liver fat by regulating the expression of enzymes involved in lipogenesis. Rats fed high GI starchy diets have more visceral adiposity, adipocyte volume, and fat pads than those on the control (low GI) diet^29^. In addition, high GI diets downregulate mRNA carnitine palmitoyltransferase 1 (CPT-1) levels in hepatocytes, thereby reducing fatty acid β-oxidation and increasing lipogenesis and fat deposition^30^. Moreover, high GL diets decrease energy expenditure and serum leptin concentrations and increase fuel storage^31^ and levels of inflammatory markers, such as C-reactive protein^32^. Therefore, through these mechanisms, starchy foods contribute to the pathogenesis of metabolic disorders. Additionally, high intake of refined grains is often accompanied by low intake of whole grains, fruits, vegetables, legumes, and dairy products^33^, which have protective effects on human metabolism^34^.

In our study, 220 g of carbohydrates from starchy foods was considered to be the optimal cut-off value for the prevention of hyperlipidemia and MetS. Based on nutritional analyses, 220 g of carbohydrates from starchy foods corresponds to 325 g (250–400 g, recommended by Chinese Food Guide Pyramid) of starchy foods, which should be consumed in the form of whole grains, tubers, and mixed beans^35^. However, this range may not be optional for individuals living in China. Therefore, it might be necessary to adapt the Chinese food guide to the dietary patterns of certain geographical areas of the country. Our results suggested that residents in Northern China should not consume more than 325 g of starchy foods.

There are some limitations to this study. First, the study population was randomly selected from a city in Northern China, which is not a representative sample of the entire Chinese population. Second, 7.9% of the participants in the original cohort study did not complete the first follow-up in 2012. Third, metabolic disorders are multifactorial conditions; while this study only evaluated dietary patterns, more detailed information is required on family history, education, and disease status. Fourth, physical activity has significant effects on dietary intake; therefore, more detailed information on energy expenditure is required. Fifth, this original study was an observational cohort study, which provides information on the association between carbohydrate consumption and risk of metabolic disorders. To assess causal relationships, experimental studies (e.g., RCTs) should be performed. Finally, a food frequency questionnaire (FFQ) was used to assess dietary patterns. While FFQs are convenient in large populations, they are not as accurate as dietary records or nutritional assessment methods that incorporate food weights.

In conclusion, high intakes of total carbohydrates and carbohydrates from starchy foods are associated with hyperlipidemia and MetS. Carbohydrate intake from starchy foods contributes more strongly to metabolic disorders. For the prevention of metabolic disorders in Northern China, the optimal daily intake of carbohydrates from starchy foods is 220 g.

## Methods

### Study population

The data were obtained from the Harbin People’s Health Study (HPHS), conducted in 2008^21^ with a follow-up in 2012 at the Harbin Medical University in Harbin, the largest city in Northern China. A total of 4,515 participants (50.5% of the whole study population) was randomly selected for the follow-up due to limited financial resources, and 4,154 (92.1%) finished the first follow-up in 2012. The participants (20–74 y of age) were randomly sampled from three communities and matched with financial status in five districts. The participants had no cancer or type 1 diabetes, and none of them were pregnant. Informed written consents were obtained from all participants. The study was reviewed by the Institutional Review Boards of the participating institutions, approved by the ethics committee of Harbin Medical University and conducted in accordance with the Declaration of Helsinki.

### Lifestyle and health data

Health exams were performed in community clinics by physicians, nurses, and medical technologists. All participants were interviewed face-to-face regarding demographic characteristics, lifestyle, physical activity, and dietary habits. Participants without self-reported diabetes underwent a 75 g oral glucose tolerance test (OGTT). Data on educational status, physical activity level, dietary intake, smoking and drinking status, and medical history were collected from all participants^21^,^36^. Participants who had smoked at least 100 cigarettes in their lifetime or smoked every day were considered to be smokers. Participants who had consumed ≥1 alcoholic beverage per day in the last 12 months prior to the survey were considered to be drinkers. Regular exercise was defined as any type of recreational or physical activity other than walking performed three or more days per week for at least 30 min. A participant who had received drug therapy for hypertension or hyperglycemia was considered to have a metabolic risk factor regardless of the laboratory data. All anthropometric indices were measured by well-trained examiners, with participants wearing light clothing and no shoes^21^.

Blood samples, collected following an overnight fast and the OGTT, were immediately centrifuged at 2,500 × *g* for 15 min. The resulting serum was stored at −80°C and used in the measurements of FBG, 2h-PG, TG, TC, HDL-C, and low density lipoprotein cholesterol (LDL-C) levels. All biochemical indices were measured in an automatic analyzer (Hitachi 7100, Tokyo, Japan). Serum insulin concentration (F-insulin, P-insulin) was determined in a ROCHE Elecsys 2010 Chemiluminescence Immune Analyzer (Roche Diagnostics). VAI was calculated using gender-specific equations^37^:

For males, VAI = (WC/(39.68 + (1.88 × BMI))) × (TG/1.03) × (1.31/HDL)

For females, VAI = (WC/(36.58 + (1.89 × BMI))) × (TG/0.81) × (1.52/HDL)

### Exposure

To assess dietary intake in the past 12 months, a FFQ was designed. The FFQ included 103 food items from 14 food groups: rice, wheat-containing foods, potato and its products, beans and its products, vegetables, fruits, livestock and its products, poultry and its products, dairy and its products, eggs and its products, fish and its products, snack, beverage, and ice cream. The FFQ has been validated in a previous study^36^. Food frequency (times/day) was multiplied by the amount of each food item consumed (g/time) to calculate the daily dietary nutrient intakes. Starchy foods included rice, wheat-containing foods, potatoes, and their products. Carb-S represented the total amount of the macronutrient from all the starchy foods^38^ (Table S2). Total energy intake was positively associated with carbohydrate intake. Therefore, carbohydrate intake was adjusted in residual models, interpreted as the composition of total carbohydrates in the diet independent of total energy intake^39^. The energy-adjusted carbohydrate intakes were applied in further analyses. Carb-O represented the difference in carbohydrate intakes between total carbohydrates and Carb-S. Starchy foods with high glycemic index (GI, >65) were analyzed with respect to MetS and hyperlipidemia risk.

### Outcomes

According to IDF criteria^21^, MetS is defined as having central obesity (WC: ≥90 cm for men and ≥80 cm for women) and two or more of the following four risk factors: high TG levels (>1.7 mmol/L), low HDL-C levels (<1.03 mmol/L in men and <1.29 mmol/L in women), high blood pressure (SBP ≥ 130 mm Hg or DBP ≥ 85 mmHg), and hyperglycemia (FBG level ≥ 5.6 mmol/L or 2h-PG level ≥ 7.8 mmol/L) or previously diagnosed T2DM. Hypertension was defined as SBP ≥ 140 mmHg or DBP ≥ 90 mmHg. T2DM was defined as FBG ≥ 7.0 mmol/L or 2h-PG ≥ 11.1 mmol/L. Hyperlipidemia was defined as TG > 2.26 mmol/L, TC > 6.22 mmol/L, HDL-C < 1.04 mmol/L, or self-reported hyperlipidemia.

In this study, the exclusion criteria were: missing data; presence of hepatitis, nephropathy, diabetes, or cardiovascular diseases; daily energy consumption <500 Kcal or >4,500 Kcal; presence of >10 blank food items, or intervention or treatment based on medical, physical, or dietary assessments. When analyzing the association between carbohydrate intake and MetS incidence, we excluded participants with MetS at baseline; therefore, only 2,734 subjects were included. Similarly, only 2,252 subjects were included in the hyperlipidemia analysis.

### Statistical analysis

Linear regressions were performed to adjust total energy consumption in residual models, with Carb-S as the outcome and total energy as the predictor. The expected value of Carb-S was calculated as the average energy intake according to the linear regressions. The sum of the expected value and the residuals represented the energy-adjusted Carb-S. We calculated quartiles of energy-adjusted Carb-S to analyze the association between Carb-S and MetS. The chi-square test and one-way ANOVA were used to test the variation in frequency and mean values of continuous variables. The results are expressed as the mean ± standard deviation. Relative risk analyses were performed using multivariate Cox’s proportional hazards regression model for risk analysis. Model 1 was derived from univariate regression analysis. In Models 2 and 3, we adjusted the demographic data and dietary risk factors possibly associated with metabolic disorders, e.g., age, sex, BMI, smoke, alcohol use, physical activities, total energy, dietary fat, and dietary fiber. Baseline levels of SBP, TC, and TG were adjusted in Model 4. Similarly, we analyzed the association between Carb-S and hyperlipidemia. This procedure was adopted in the analysis of total carbohydrates, Carb-O, high GI starchy foods and medium or low GI starchy foods. Receiver operating characteristics (ROC) analysis was used to identify optimal cut-off values for Carb-S; the maximum Youden index (sensitivity + specificity −1) was used to identify MetS and dyslipidemia. All statistical analyses were performed with SAS software (version 9.1; SAS Institute, Inc., Cary, NC, USA). *P* values were 2-tailed; *P* < 0.05 was considered to be statistically significant.

## Additional Information

**How to cite this article**: Feng, R. *et al.* High carbohydrate intake from starchy foods is positively associated with metabolic disorders: a Cohort Study from a Chinese population. *Sci. Rep.* **5**, 16919; doi: 10.1038/srep16919 (2015).

## Supplementary Material

Supplementary Information

## Figures and Tables

**Table 1:** The basic characteristics of all participants after quartering intake of carbohydrate from starchy foods in energy-adjusted residual model after excluding all metabolic syndrome patients in 2008.

	Energy-adjusted carbohydrate intake from starchy food (g)				*P*
	Q1	Q2	Q3	Q4	
Participants (n)	682	684	682	686	
Age (y)	51.86 ± 11.64	54.36 ± 10.36	54.90 ± 9.86	55.98 ± 10.58	<0.01
Male (%)	25.15	27.49	32.26	40.64	<0.01
Education (%)					
None	2.57	2.54	1.31	2.18	<0.01
Primary	1.93	3.49	5.56	7.48	
Junior high school	26.69	30.48	32.68	35.20	
Senior high school	31.51	33.02	34.97	36.14	
College	36.66	29.84	24.51	18.38	
Postgraduate and above	0.64	0.63	0.98	0.62	
Physical activity (%)					
Light	85.89	83.58	86.21	82.57	0.13
Middle	13.81	16.12	12.85	15.29	
Heavy	0.30	0.30	0.94	2.14	
Alcohol (%)	38.01	39.18	39.12	39.47	0.98
Smoking (%)	11.76	12.39	17.70	17.70	0.03
BMI (kg/m^2^)	24.19 ± 3.47	24.29 ± 3.22	24.11 ± 2.82	24.52 ± 3.32	0.37
WC (cm)	80.78 ± 10.17	81.35 ± 9.18	81.49 ± 8.61	82.63 ± 9.47	0.08
SBP (mmHg)	123.94 ± 17.89	127.84 ± 19.5	127.53 ± 19.56	128.67 ± 20.32	0.01
DBP (mmHg)	76.01 ± 10.37	77.79 ± 10.92	77.1 ± 10.56	77.53 ± 10.36	0.13
FBG (mmol/L)	4.76 ± 1.28	4.76 ± 1.20	4.87 ± 1.39	4.88 ± 1.57	0.49
2h-PG (mmol/L)	5.79 ± 2.85	5.87 ± 2.52	6.15 ± 3.39	6.08 ± 3.07	0.35
F-insulin (IU/mL)	7.66 ± 4.60	7.42 ± 4.32	6.85 ± 4.53	6.93 ± 4.43	0.15
P-insulin (IU/mL)	32.74 ± 26.15	34.84 ± 32.66	32.92 ± 28.17	34.37 ± 30.18	0.80
HOMA-IR	1.58 ± 0.98	1.54 ± 0.92	1.44 ± 0.95	1.44 ± 0.96	0.27
TC (mmol/L)	4.74 ± 0.86	4.91 ± 0.93	4.92 ± 1.07	4.8 ± 0.88	0.04
TG (mmol/L)	1.30 ± 0.79	1.49 ± 1.23	1.58 ± 1.23	1.50 ± 0.98	0.01
HDL-C (mmol/L)	1.32 ± 0.32	1.32 ± 0.30	1.30 ± 0.32	1.31 ± 0.32	0.84
Obesity (%)	11.50	12.39	7.94	11.99	0.23
Central obesity (%)	36.87	37.54	37.76	39.71	0.89
Hypertension (%)	21.99	30.12	31.47	29.53	0.03
Hyperlipidemia (%)	29.12	34.41	34.22	33.53	0.41

**Table 2:** The risk ratios (RRs) of hyperlipidemia and metabolic syndrome for quartiles of energy-adjusted carbohydrate intake from starchy foods at baseline.

	Cases/N	Model 1	Model 2	Model 3	Model 4
Hyperlipidemia					
*P* for trend		0.03	0.04	0.02	0.02
Q1	97/562	1.00	1.00	1.00	1.00
Q2	104/564	1.14(0.80,1.62)	1.04(0.72,1.50)	1.12(0.76,1.65)	1.45(0.91,2.31)
Q3	118/564	1.47(1.03,2.10)	1.35(0.94,1.95)	1.43(0.97,2.10)	1.91(1.13,3.23)
Q4	126/562	1.63(1.15,2.32)	1.42(0.99,2.05)	1.47(1.10,2.15)	1.73(1.05,3.35)
MetS					
*P* for trend		<0.01	0.01	0.01	<0.01
Q1	75/682	1.00	1.00	1.00	1.00
Q2	78/684	1.05(0.73,1.50)	1.00(0.69,1.45)	0.93(0.66,1.38)	1.01(0.66,1.55)
Q3	116/682	1.81(1.29,2.55)	1.82(1.27,2.60)	1.72(1.19,2.49)	1.89(1.19,3.02)
Q4	100/686	1.44(1.02,2.04)	1.31(0.91,1.87)	1.24(0.95,1.80)	1.48(1.01,2.69)

Model 1 has not been adjusted by any potential factors; Model 2 has been adjusted by age, sex, BMI; Model 3 has been adjusted by age, sex, BMI, smoke, drink, physical activities, total energy, fat and fiber; Model 4 has been adjusted by all factors in Model 3 and SBP, TC and TG.

**Table 3:** The associations between energy-adjusted total carbohydrate intake and metabolic diseases, including hyperlipidemia and MetS.

	Cases/N	Model 1	Model 2	Model 3	Model 4
Hyperlipidemia					
*P* for trend		0.03	0.03	<0.01	<0.01
Q1	118/562	1.00	1.00	1.00	1.00
Q2	107/564	0.85(0.60,1.20)	0.85(0.59,1.21)	1.07(0.71,1.60)	1.45(0.92,2.29)
Q3	103/564	0.84(0.59,1.19)	0.84(0.59,1.21)	1.63(0.88,2.32)	1.73(0.98,3.05)
Q4	117/562	1.30(1.15,1.46)	1.25(1.06,1.47)	2.50(1.20,5.22)	3.05(1.25,7.45)
MetS					
*P* for trend		0.02	0.02	0.01	<0.01
Q1	90/682	1.00	1.00	1.00	1.00
Q2	86/684	0.93(0.66,1.31)	0.96(0.67,1.37)	1.12(0.75,1.68)	1.16(0.76,1.76)
Q3	98/682	1.11(0.79,1.55)	1.06(0.75,1.51)	1.51(0.94,2.42)	1.59(0.95,2.65)
Q4	95/686	1.28(1.11,1.48)	1.31(1.18,1.46)	2.01(0.99,4.08)	2.24(1.00,5.03)

Model 1 has not been adjusted by any potential factors; Model 2 has been adjusted by age, sex, BMI; Model 3 has been adjusted by age, sex, BMI, smoke, drink, physical activities, total energy, fat and fiber; Model 4 has been adjusted by all factors in Model 3 and SBP, TC and TG.

**Table 4:** The risk ratios (RRs) of metabolic disorders for energy-adjusted carbohydrate from diet excluded those from starchy foods.

	Cases/N	Model 1	Model 2	Model 3	Model 4
Hyperlipidemia					
*P* for trend		0.07	0.06	0.12	0.14
Q1	127/562	1.00	1.00	1.00	1.00
Q2	111/564	0.76(0.53,1.08)	0.79(0.55,1.12)	0.85(0.59,1.24)	0.87(0.58,1.31)
Q3	99/564	0.65(0.45,0.92)	0.66(0.46,0.95)	0.71(0.48,1.05)	0.68(0.42,1.08)
Q4	108/562	0.70(0.49,0.99)	0.77(0.54,1.11)	0.85(0.56,1.29)	0.54(0.29,1.20)
MetS					
*P* for trend		0.06	0.06	0.11	0.13
Q1	105/682	1.00	1.00	1.00	1.00
Q2	91/684	0.81(0.58,1.13)	0.82(0.58,1.16)	0.85(0.59,1.22)	0.86(0.59,1.27)
Q3	95/682	0.85(0.61,1.18)	0.81(0.57,1.14)	0.87(0.60,1.26)	0.88(0.58,1.35)
Q4	78/686	0.67(0.47,0.94)	0.67(0.47,0.96)	0.71(0.47,1.08)	0.71(0.41,1.24)

Model 1 has not been adjusted by any potential factors; Model 2 has been adjusted by age, sex, BMI; Model 3 has been adjusted by age, sex, BMI, smoke, drink, physical activities, total energy, fat and fiber; Model 4 has been adjusted by all factors in Model 3 and SBP, TC and TG.

**Table 5:** The anthropometric measurements and biochemical indexes changes during 4.2-year following-up in all participants after quartering energy-adjusted carbohydrate from starchy foods.

	Energy-adjusted carbohydrate from starchy food (g)				*P*
	Q1	Q2	Q3	Q4	
BMI (kg/m^2^)	−0.39 ± 3.43	−0.49 ± 3.19	−0.05 ± 3.07	−0.19 ± 2.67	0.33
WC (cm)	2.13 ± 10.28	2.13 ± 10.32	1.83 ± 8.6	2.21 ± 8.93	0.97
SBP (mmHg)	−1.47 ± 20.53	0.14 ± 19.15	−0.63 ± 20.68	−1.58 ± 21.69	0.73
DBP (mmHg)	−2.40 ± 11.74	−1.94 ± 10.66	−0.45 ± 11.76	−1.96 ± 10.62	0.19
FBG (mmol/L)	−0.12 ± 1.37	−0.12 ± 1.78	0.07 ± 1.57	0.00 ± 1.80	0.43
2h-PG (mmol/L)	0.30 ± 2.44	0.40 ± 3.59	0.29 ± 3.51	0.57 ± 3.13	0.76
F-insulin (IU/mL)	0.22 ± 6.11	0.7 ± 5.99	1.1 ± 6.27	0.48 ± 6.24	0.57
P-insulin (IU/mL)	9.09 ± 37.47	5.3 ± 38.71	8.00 ± 33.62	8.75 ± 35.49	0.76
HOMA-IR	0.03 ± 1.2	0.15 ± 1.23	0.21 ± 1.32	0.07 ± 1.13	0.51
VAI	0.56 ± 2.38	0.98 ± 3.3	1.26 ± 3.71	1.13 ± 2.64	0.05
TC (mmol/L)	0.49 ± 1.06	0.44 ± 1.05	0.49 ± 0.94	0.44 ± 0.94	0.86
TG (mmol/L)	0.24 ± 1.61	0.32 ± 1.26	0.55 ± 1.88	0.40 ± 1.22	0.04
HDL-C (mmol/L)	−0.05 ± 0.41	−0.11 ± 0.41	−0.12 ± 0.41	−0.16 ± 0.42	0.01

**Table 6:** Sensitivity, specificity, and Youden index for cut-off values of intake of carbohydrate from starchy foods to identify hyperlipidemia and MetS.

Cut-off	Sensitivity	Specificity	Youden Index
Hyperlipidemia			
220-0.2SD	70.11	36.77	6.88
220-0.1SD	67.19	41.03	8.22
220	64.27	47.78	12.05
220+0.1SD	59.10	52.22	11.32
220+0.2SD	53.71	55.60	9.30
MetS			
230-0.2SD	44.44	62.65	7.10
230-0.1SD	37.94	66.02	3.96
230	64.23	49.18	13.41
230+0.1SD	25.47	76.12	1.60
230+0.2SD	23.04	79.39	2.42
