# Supplementary material for: High carbohydrate intake from starchy foods is positively associated with metabolic disorders: a Cohort Study from a Chinese population
Source: Sci Rep. 2015 Nov 19;5:16919. doi: 10.1038/srep16919 (PMC4652281; doi:10.1038/srep16919)
Supplement: Supplementary Information [file srep16919-s1.pdf]

High carbohydrate intake from starchy foods is positively associated with metabolic disorders: a Cohort Study from a Chinese population

Rennan Feng*, Shanshan Du*, Yang Chen*, Sining Zheng, Wei Zhang, Guanqiong Na, Ying Li & Changhao Sun

Table S1. The risk ratios (RRs) of metabolic disorders for high or medium and low GI starchy food.

	Cases/n	Model 1	Model 2	Model 3	Model 4
**High GI Food**					
Hyperlipidemia					
Q1	100/562	1.00	1.00	1.00	1.00
Q2	110/564	1.12(0.79,1.61)	1.01(0.70,1.46)	0.96(0.65,1.42)	1.01(0.67,1.50)
Q3	104/564	1.01(0.71,1.45)	0.94(0.65,1.36)	0.91(0.61,1.35)	0.99(0.65,1.51)
Q4	124/562	1.37(1.09,2.24)	1.30(1.02,1.88)	1.37(1.08,2.24)	1.32(1.03,2.17)
MetS					
Q1	77/682	1.00	1.00	1.00	1.00
Q2	89/684	1.23(0.86,1.75)	1.14(0.79,1.65)	1.05(0.72,1.55)	1.06(0.71,1.57)
Q3	91/682	1.24(0.87,1.76)	1.19(0.82,1.71)	1.14(0.77,1.67)	1.16(0.78,1.75)
Q4	105/686	1.51(1.07,2.14)	1.46(1.02,2.09)	1.47(1.02,2.11)	1.57(1.01,2.42)
**Medium and low GI Food**					
Hyperlipidemia					
Q1	117/562	1.00	1.00	1.00	1.00
Q2	95/564	0.74(0.52,1.06)	0.70(0.48,1.06)	0.69(0.47,1.03)	0.71(0.49,1.09)
Q3	114/564	1.03(0.72,1.46)	0.94(0.65,1.35)	0.87(0.59,1.28)	0.89(0.60,1.31)
Q4	112/562	1.02(0.71,1.45)	0.94(0.65,1.35)	0.89(0.61,1.31)	0.93(0.63,1.37)
MetS					
Q1	103/682	1.00	1.00	1.00	1.00
Q2	86/684	0.80(0.57,1.13)	0.73(0.51,1.04)	0.71(0.50,1.06)	0.69(0.45,1.02)
Q3	80/682	0.75(0.54,1.10)	0.70(0.50,1.03)	0.69(0.49,1.02)	0.70(0.49,1.03)
Q4	93/686	0.88(0.63,1.23)	0.80(0.57,1.14)	0.75(0.52,1.08)	0.76(0.53,1.11)

Model 1 has not been adjusted by any potential factors; Model 2 has been adjusted by age, sex, BMI; Model 3 has been adjusted by age, sex, BMI, smoke, drink, physical activities, total energy, fat and fiber; Model 4 has been adjusted by all factors in Model 3 and SBP, TC and TG.

Table S2 Food items in current study.

Food Groups	Food Items
Starchy foods	
Rice	Rice, milet, corn
Wheaten foods	Cookie, cake, steamed buns, noodle, bread
Potato and its products	White potato, sweet potato, vermicelli
Other foods	
Beans	Beans, beancurd, soybean milk, uncongealed beancurd
Vegetables	Turnip, carrot, spinage, bean sprout, eggplant, tomato, green bean, pepper, winter melon, cucumber, pumkin, garlic, shallot, onion, onion chives, cabbage, sauerkraut, agaric, cauliflower, celery, lettuce, mushroom, kelp
Fruits	Apple, jujude, persimmon, pear, orange, peach, grape, pomegranate, strawberry, kiwi fruit, pineapple, litchi, mango, pawpaw, watermelon, honey melon, mushmelon
Livestock	Pork, beef, mutton, sausage, liver and internal organs
Poultry	Chicken, duck, goose
Dairy	Milk, yoghourt, milk power
Eggs	Eggs
Fish	Fish, shellfish, mollusc
Snack	Preserved, biscuit, chocolate, peanut, melon seed, nuts
Beverage	Carbonated beverage, fruit juice, tea, coffee
Ice cream	Ice cream
